# Multilevel analysis of factors associated with unmet need for family planning among Malawian women

**DOI:** 10.1186/s12889-020-08885-1

**Published:** 2020-05-15

**Authors:** Owen Nkoka, Watanja M. Mphande, Peter A. M. Ntenda, Edith B. Milanzi, Victor Kanje, Shiaau J. G. Guo

**Affiliations:** 1 Institute for Health Research and Communication (IHRC), P.O Box 1958, Lilongwe, Malawi; 2 School of Public Health, College of Public Health, Taipei Medical University, 250 Wuxing Street, Xinyi Taipei, Taiwan 110; 3 Ministry of Health, P.O. Box 30377, Lilongwe, 3 Malawi; 4 University of Malawi, College of Medicine, Malaria Alert Centre, Private Bag 360, Chichiri, Blantyre 3, Malawi

**Keywords:** Unmet need, Family planning, Multilevel, Malawi

## Abstract

**Background:**

Malawi has a high fertility rate which is also characterized by a relatively high prevalence of unmet need for contraception. However, little is known about the influence of individual- and community- level characteristics on unmet need in Malawi. This study examined the individual- and community- level factors associated with unmet need for family planning (FP) among Malawian women.

**Methods:**

Data from the 2015–16 Malawi demographic and health survey were used to analyze 15, 931 women. The association between individual- and community- level factors and unmet need was assessed using multilevel binary logistic regression models.

**Results:**

The prevalence of total unmet need was 21.0%. Women aged ≥35 years were more likely to have total unmet need [adjusted odds ratio (aOR) = 1.19, 95% confidence interval (CI) = 1.04–1.35] compared with those aged 15–24 years. Women who were married [aOR = 0.41, 95% CI = 0.35–0.48], and those employed [aOR = 0.78, 95% CI = 0.71–0.85] were associated with less likelihood of having total unmet need compared with unmarried, and unemployed women, respectively. At community-level, women from communities with a high percentage of women from rich households [aOR = 0.81, 95% CI = 0.67–0.96], and those from communities with a middle and high percentage of educated women [aOR = 0.86, 95% CI = 0.76–0.96 and aOR = 0.81, 95% CI = 0.70–0.93, respectively] were less likely to have total unmet need for FP compared with those from communities with low percentages of rich and educated women, respectively. The proportional change in variance showed that about 36.0% of total variations in the odds of unmet need across the communities were explained by both individual- and community-level factors. Moreover, the intraclass correlation showed that about 3.0% of the total variation remained unexplained even after controlling for both individual- and community-level factors.

**Conclusion:**

Both individual- and community- level factors influenced unmet need for FP in Malawi. Public health practitioners should conduct community profiling and consider individual and community factors when designing FP programs.

## Background

The use of contraceptives to regulate fertility either for child spacing or limiting childbearing has essential health benefits [1]. For instance, appropriate child spacing (i.e., 2 years or more) has been associated with a reduced likelihood of preterm births, which is a key contributor to neonatal and infant mortality [2]. Globally, approximately 50 million women with either mistimed or unwanted pregnancies have an induced abortion, as a way of fertility control, most of which are unsafe resulting in high maternal mortality [3]. Thus, family planning (FP) through the use of modern contraceptives is of public health importance. However, gaps in terms of meeting the demand for FP services in developing countries exist. Approximately 230 million women in developing countries had an unmet need for modern FP methods in 2019 [4], an increase from the 225 million estimates reported in 2014 [5].

Women are considered to have an unmet need for FP if they want to stop or delay/postpone childbearing but are not using any method of contraception [5]. Unmet need for FP is an important indicator for the gap in terms of women’s reproductive intentions and their contraceptive behavior [6]. Additionally, the unmet need for FP is important for the assessment of the progress towards achieving universal access to sexual and reproductive health services [7]. There are two types of unmet need; unmet need for spacing and limiting. Unmet need for spacing refers to a situation where a woman wants to postpone/delay pregnancy while limiting is when the woman wants no more children and is not using any contraception [8].

Malawi has made tremendous efforts to improve the accessibility of FP services by investing in human resources and training, deployment of lower cadres of health professionals to provide community services and expanding mobile and outreach services [9]. Therefore, commendable strides have been made in reducing unmet need from 35.0% in 1992 to 26.0% in 2010 [10]. However, the 26.0% prevalence of unmet need in Malawi is relatively higher compared to the prevalence of unmet need in Rwanda (19.0%) reported in the same year (i.e., 2010) [11]. Moreover, compared with Nigeria’s 10.8% rate of unintended pregnancies, a relatively high rate (47.0%) of unintended pregnancies in Malawi has been reported thus underscoring that unmet need for FP may be a persistent problem among Malawian women [12]. Additionally, Malawi’s maternal mortality rate is one of the highest in sub-Saharan Africa at 510 deaths per 100,000 live births. Some of the leading causes of maternal mortality include unsafe abortions resulting from unwanted pregnancies, high fertility rates and teenage pregnancies [13]. Therefore, improving access to modern FP methods is crucial in Malawi.

Previous studies in other countries have assessed factors associated with unmet need for FP such as maternal educational level, and maternal age [14]. Additionally, other studies reported that discussing FP issues with a husband, and receiving partner support reduced the likelihood of having unmet need for FP [15, 16]. An Ethiopian study revealed that unemployed women were more likely to have unmet need for FP than their employed counterparts [17]. However, inconsistent findings have been reported in different settings suggesting the need for setting-specific data on the factors associated with unmet need. For example, while the area of residence was associated with total unmet need in Burundi [14], no significant association was observed in Ghana [18]. Additionally, there is a paucity of data on the contextual influences on unmet need. Community characteristics influence the access to, and the utilization of health services [19]. Considering the effects of community characteristics may help account for the differences observed in literature regarding the factors associated with unmet need for FP. Results may also help public health practitioners working in FP programs to design tailored FP interventions.

Therefore, the objective of this study was to investigate individual- and community-level factors associated with unmet need in Malawian women, utilizing data from a nationally representative sample.

## Methods

### Study design

This was a cross-sectional study conducted using secondary data from the 2015–16 Malawi demographic health survey (MDHS). A detailed explanation of the methodology of the MDHS has been outlined elsewhere [20]. In brief, the survey used a two-stage cluster sampling method in which the first stage, clusters were randomly selected from the sampling frame (i.e. the 2008 Malawi population and housing census) and household listing. The second stage involved a systematic selection of households from the selected clusters.

### Study setting

Malawi is located in southern-central Africa and has a population of approximately 17.5 million people [21]. Precisely, there has been a 35.0% population growth rate since the last census was conducted in 2008 with the population expected to double by 2042 (based on the current annual growth rate of 2.9) [21]. As of 2018, a majority (84.0%) of Malawians were rural dwellers. In terms of contraceptive use, the use of modern contraceptive methods among married women of reproductive age in Malawi increased from 28, 58, and 59.2% in 2004, 2015, and 2016, respectively [21, 22], with the sustained use of injectable contraceptives and of long-acting and permanent methods of contraception. The observed increase has been attributed to high level commitment, continued health financing, expanded and innovative service delivery options [10]. Unmet need is high in Malawi, with 22% of married women—and 52% of unmarried but sexually active women—aged 15–19 having an unmet need for family planning [23]. Malawi has created an enabling policy environment to increase the utilization of FP services with a special focus on adolescent women. So far, the following achievements have been made regarding FP policies in Malawi: (1) the establishment of national FP-related policies (such as National Reproductive Health Service Delivery Guidelines [24], National Sexual and Reproductive Health and Rights Policy [25], and the National Population Policy [26]); (2) ensuring that these policies address some of the barriers to accessing FP services such as age or marital status restrictions; (3) harnessing the prevention of rights violations and practices that have a broad harmful effect on vulnerable groups (e.g., adolescent women); (4) integrating the FP policies with other youth-related policies such as the National Youth Policy [27]; (5) addressing the contextual factors that influence adolescent access to information and services; (6) and ensuring consistent implementation of the FP policies through the establishment of accountability and necessary data collection mechanisms [23].

Data collection and sample size.

Face to face interviews using pre-tested questionnaires were conducted by experienced and trained data collectors. Information on sociodemographic, health-related factors and use of contraceptives was collected. To assess unmet need for FP, a number of questions related to fertility intentions were asked in the MDHS women’s questionnaire (e.g., “Are you pregnant now?”, “When you got pregnant, did you want to get pregnant at that time?”, “Would you like to have (a/another) child, or would you prefer not to have any (more) children?”). Detailed questions asked to calculate unmet need for FP can be obtained from the MDHS report published elsewhere [20]. A total of 24,562 out of 25,146 eligible women were interviewed, representing a 98.0% response rate. The current analysis was restricted to fecund women who were married/living with a partner or unmarried but sexually active (*n* = 15, 931). Women who were infecund, and sexually inactive were excluded from the final analysis.

### Measures

#### Outcome measure

Based on the revised definition of unmet need for FP by Bradley et al. [28], we calculated unmet need for spacing by coding “1” to; (a) women who were not using contraception and were pregnant or postpartum amenorrhoeic (last period not returned since last live birth in the last 2 years) but wanted current pregnancy/last birth later and, (b) women who were not pregnant or postpartum amenorrhoeic who were not using contraception but reported wanting to have a child in the next ≥2 years, or wanted to have children but had undecided timing or those that were undecided if they wanted a child. Therefore, women who reported to have been using contraception for spacing or with no unmet need were coded as “0”.

Similarly, unmet need for limiting was calculated by coding “1” to; (a) pregnant women or postpartum amenorrhoeic women (last period not returned since last live birth in the last 2 years) but did not want current pregnancy/last birth at all, and (b) women who were not pregnant or postpartum amenorrhoeic who were not using any contraceptive but reported wanting no more children. Those that reported using contraception for limiting or with no unmet need were coded “0”.

Total unmet need was a dichotomous variable calculated by combining unmet need for spacing or limiting. Women with unmet need for spacing or limiting were coded as “1” while those using contraception for spacing or limiting or with no unmet need were coded as “0”.

#### Independent variables

Independent variables were assessed at two-levels; level 1 included the individual-level variables while level 2 consisted of community/contextual factors.

Individual-level variables were considered based on their importance in literature [8, 14, 18] and included; sociodemographic factors such as woman’s age in years (15–24, 25–34, ≥ 35), marital status (married, unmarried) wealth (calculated using principal component analysis in which the scores obtained from ownership of household items were grouped as poor (lower 40%), middle (middle 20%), and rich (upper 40%)), employed (yes or no), residence (urban, rural), region (northern, central, southern), women’s educational level was defined as the level of schooling ever attended [20] (no formal education, primary, secondary and higher). Fertility intention drivers such as the number of children ever had (0, 1, 2+), whether the women ever experienced the death of child (yes or no), religion was categorized as “Catholics”, “protestants”, and “Muslims and other”, and media exposure (those who reported reading newspapers, listening to the radio, or watching television at least once a week were coded as “yes” or otherwise as “no”). Perceived distance to the health facility, categorized as “problem” or “no problem”, was included an access-related factor.

To analyze variables at community-level, aggregation of four key sociodemographic factors and one access-related factor from individual-level to community-level was done. These factors were selected based on their importance in previous studies [29, 30]. A community was defined as the primary sampling unit (i.e., cluster) of the MDHS survey. Community wealth, employment, women’s education, partner education, and distance to health facility were defined as the proportion of rich, employed, women with any education, and women who perceived distance to health facility as a problem within a community. For easy interpretation, the percentages were categorized into three levels using tertiles (low, middle, and high).

### Statistical analysis

#### Distribution of study participants’ characteristics

All analyses were performed using Stata version 15.0 (Stata Corp LP, College Station, TX, USA). The “svy” command was used to take into account the sampling weights and adjust for clustering effects of the hierarchical nature of the MDHS data. Distribution of study participants’ characteristics according to their unmet need status was assessed using Chi square tests. The total demand for FP was calculated by adding the percentage of women with unmet need and the percentage of those using any contraception method.

#### Modeling approaches

Three modelling approaches were adopted namely; fixed effect, random effects, and the goodness of fit assessments.

##### Fixed effects

First, a two-level multilevel binary logistic regression model was fitted, using “*xtmelogit*” command in Stata, to assess the association of individual- and community-level factors, and the total unmet need. Women (level 1) were clustered within their communities (level 2). Four models were tested; a null model which was the unconditional model included the outcome variable(s) only to assess the variance in unmet need between communities, model I included outcome and individual-level variables, model II included outcome and community-level variables, and model III included the outcome variables, and both individual- and community- level variables. The fixed effects for the multilevel binary logistic regression model were reported as adjusted odds ratios (aORs) with 95% confidence intervals (CI).

##### Random effects

The “*xtmelogit*” command allowed for the assessment of random effects at the community level. Measures of variation (random effects) were assessed using several indicators such as area variance (AV) with 95% CI, the intraclass correlation coefficient (ICC), proportional change in variance (PCV), and the median odds ratio (MOR).

##### Goodness of fit

The goodness-of-fit of each model was assessed using the Akaike information criterion (AIC), with a lower value representing a closer model fit. The variance inflation factor (VIF) was used to assess multicollinearity. None of the variables displayed multicollinearity problems (all VIF < 10) (Table S1).

### Sensitivity analyses

In sensitivity analyses, we repeated the main analyses by excluding subsamples of the study population to examine the effect on our results. First, women that were not using contraceptives but were pregnant or postpartum amenorrhoeic and reported to have wanted current pregnancy/last birth, or those that were not using any contraception and were not pregnant or postpartum amenorrhoeic but reported to have wanted a child within 2 years were regarded as having “no unmet need”. These were then excluded from the analyses. Second, we repeated the analysis excluding those that were using traditional methods to assess the factors associated with unmet need for modern FP methods. Third, the analysis was restricted to married women to control for factors such as age at first marriage and partner’s educational level.

### Ethics statement

Permission to utilize the data was obtained from the demographic health survey program. The survey protocol was reviewed and approved by the National Health Sciences Research Board of Malawi, Institutional Review Board (IRB) of ICF Macro, and Centers for Disease Control and Prevention (CDC) in Atlanta. Informed consent was obtained at the beginning of each interview by the MDHS data collectors.

## Results

Data for 15, 931 sexually active women (15, 110 married and 821 unmarried) (level 1) nested within 850 communities (level 2) were analyzed. The overall prevalence of unmet need among the total sample was 21.0% (3350). The prevalence of unmet need for spacing and limiting were 12.6 and 8.4%, respectively.

**Fig. 1:**
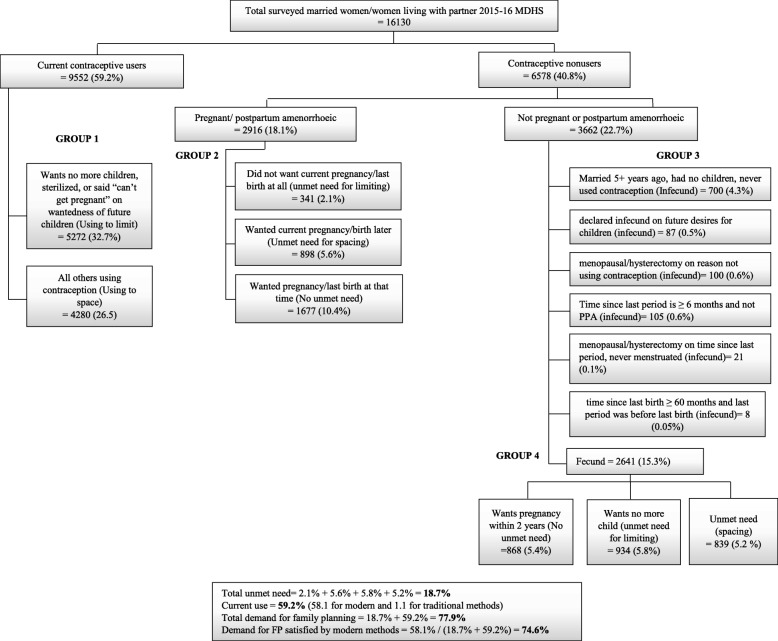
Unmet need among married women in Malawi, 2015–16 (Bradley et al. [28])

Specifically, among married women, the prevalence of unmet need for FP was 18.7% while for contraceptive use was 59.2% (58.1% for modern and 1.1% for traditional methods). The total demand for FP was 77.9% while the proportion of demand satisfied by modern methods was 74.6% (Fig. 1).

**Fig. 2:**
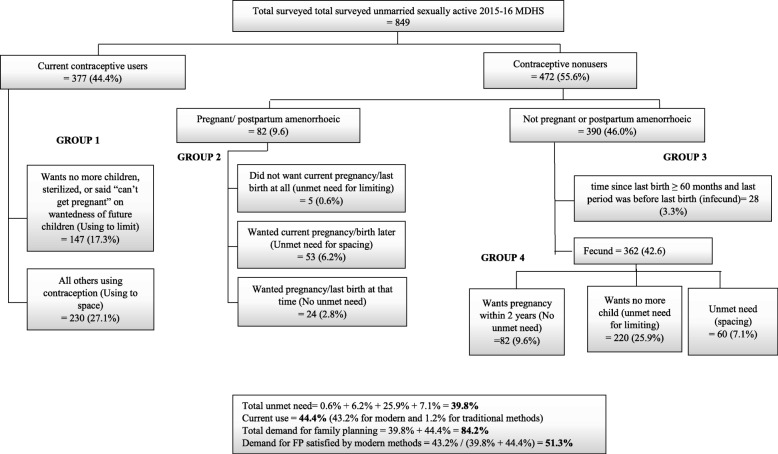
Unmet need among unmarried 2015–16 (Bradley et al. [28])

Among unmarried sexually active women, the prevalence of contraceptive use was 39.8% (43.2% for modern and 1.2% for traditional methods). The total demand for FP was 84.2% while the proportion of demand satisfied by modern methods was 51.3% (Fig. 2).

### Distribution of participants’ characteristics according to unmet need status

The distribution of study participants’ characteristics according to unmet need (dichotomous variable) are listed in Table 1. Higher proportions of women with unmet need were observed among women who were younger (22.1%), with no child (29.6%), from poor households (27.1%), unemployed (25.2%), unmarried (41.1%), from rural areas (21.4%), those who belonged to Islam and other religions (22.5%), who had no media exposure (22.6%), from communities with a low percentage of employed women (24.5%), from communities with a low percentage of educated women (23.5).

**Table 1:** Distribution of study participants according to unmet need status among sexually active women

Variable	Unmet need – (*n* = 15,931)		
	No (*n* = 12,581)	Yes (*n* = 3350)	*p*-value ^a^
*Individual-level factors*			
Woman’s age (years)			**0.011**
15–24	4123 (77.9)	1172 (22.1)	
25–34	5014 (80.6)	1209 (19.4)	
≥ 35	3444 (78.0)	969 (21.9)	
Number of children ever had			**< 0.001**
0	348 (70.4)	357 (29.6)	
1	2221 (80.3)	546 (19.7)	
2+	9512 (79.5)	2447 (20.5)	
Wealth			**0.049**
Poor	4918 (77.9)	1397 (27.1)	
Middle	2432 (79.0)	649 (21.0)	
Rich	5230 (80.0)	1304 (20.0)	
Marital status			**< 0.001**
Unmarried	483 (58.9)	338 (41.1)	
Married	12,098 (80.1)	3012 (19.9)	
Employed			**< 0.001**
No	3342 (74.7)	1129 (25.2)	
Yes	9239 (80.6)	2221 (19.4)	
Residence			**0.046**
Urban	2195 (80.9)	518 (19.1)	
Rural	10,386 (78.6)	2832 (21.4)	
Region			**< 0.001**
Northern	1473 (75.8)	471 (24.2)	
Central	5691 (82.1)	1239 (17.9)	
Southern	5417 (76.8)	1640 (23.2)	
Woman’s educational level			0.216
No formal education	1571 (77.0)	470 (23.0)	
Primary	8108 (79.2)	2135 (20.8)	
Secondary and higher	2902 (79.6)	745 (20.4)	
Religion			**0.001**
Catholics	2556 (81.1)	596 (18.9)	
Protestants	3018 (80.8)	717 (19.2)	
Muslims and other	7007 (77.5)	2037 (22.5)	
Experienced death of child			**< 0.011**
No	11,642 (79.7)	2968 (20.3)	
Yes	939 (71.1)	382 (28.9)	
Media exposure			**< 0.001**
No	7579 (77.4)	2213 (22.6)	
Yes	5002 (81.5)	1137 (18.5)	
Distance to HF			0.084
No problem	5610 (79.8)	1421 (20.2)	
Problem	6971 (78.3)	1929 (21.7)	
*Community-level factors*			
Community wealth			0.051
Low	5063 (77.9)	1433 (22.1)	
Middle	4158 (78.7)	1124 (21.3)	
High	3360 (80.9)	793 (19.1)	
Community employment			**< 0.001**
Low	3631 (75.5)	1179 (24.5)	
Middle	4075 (79.4)	1058 (20.6)	
High	4875 (81.4)	1113 (18.6)	
Community women’s education			**< 0.001**
Low	4435 (76.5)	1365 (23.5)	
Middle	4557 (80.2)	1122 (19.8)	
High	3589 (80.6)	863 (19.4)	
Community distance to HF			0.115
Low	3263 (80.7)	779 (19.3)	
Middle	4889 (78.2)	1366 (21.8)	
High	4429 (78.6)	1205 (21.4)	

^a^*p*-value from chi square test, bold means significant at *p* < 0.05

### Modeling approaches (fixed effects)

#### Factors associated with total unmet need

Table 2 displays the measures of association from the multilevel binary logistic regression model. Results from model III which accounted for both individual- and community- level variables revealed that married women [aOR = 0.41, 95% CI = 0.35–0.48], employed [aOR = 0.78, 95% CI = 0.71–0.85], from the central and southern regions [aOR = 0.69, 95% CI = 0.60–0.80 and aOR = 0.80, 95% CI = 0.70–0.92, respectively], with media exposure [aOR = 0.82, 95% CI = 0.75–0.90], from communities with a high percentage of women from rich households [aOR = 0.81, 95% CI = 0.67–0.96], from communities with a high percentage of employed women [aOR = 0.88, 95% CI = 0.78–0.99], from communities with a middle and high percentage of educated women [aOR = 0.86, 95% CI = 0.76–0.96 and aOR = 0.81, 95% CI = 0.70–0.93, respectively] were less likely to have total unmet need for FP compared with their defined counterparts. Conversely, women aged ≥35 years were more likely [aOR = 1.19, 95% CI = 1.04–1.35] to have total unmet need for FP compared with those aged 15–24 years. Additionally, compared with Catholic women, women belonging to Islam and other religions were more likely [aOR = 1.12, 95% CI = 1.01–1.25] to have total unmet need for FP. Women from communities with a middle percentage of women complaining about the distance to health facility were more likely [aOR = 1.17, 95% CI = 1.03–1.32] to have unmet need for FP compared with those from communities with a low percentage of women complaining about the distance to health facility.

**Table 2:** Multilevel logistic analysis of factors associated with total unmet need among sexually active women

Variable	Null model	Model I aOR (95% CI)	Model II aOR (95% CI)	Model III aOR (95% CI)
*Individual-level factors*				
Woman’s age (years)				
15–24		1.00		1.00
25–34		0.98 (0.87–1.10)		0.98 (0.87–1.10)
≥ 35		**1.17 (1.03–1.33)**		**1.19 (1.04–1.35)**
Number of children ever had				
0		1.00		1.00
1		0.61 (0.37–1.01)		0.60 (0.36–1.00)
2+		0.62 (0.37–1.04)		0.61 (0.36–1.03)
Wealth				
Poor		1.00		1.00
Middle		0.94 (0.84–1.05)		0.95 (0.85–1.06)
Rich		0.94 (0.84–1.05)		0.99 (0.88–1.11)
Marital status				
Unmarried		1.00		1.00
Married		**0.42 (0.35–0.49)**		**0.41 (0.35–0.48)**
Employed				
No		1.00		1.00
Yes		**0.76 (0.69–0.82)**		**0.78 (0.71–0.85)**
Residence				
Urban		1.00		1.00
Rural		1.09 (0.96–1.25)		0.91 (0.77–1.07)
Region				
Northern		1.00		1.00
Central		**0.78 (0.69–0.89)**		**0.69 (0.60–0.80)**
Southern		0.91 (0.81–1.03)		**0.80 (0.70–0.92)**
Woman’s educational level				
No formal education		1.00		1.00
Primary		**0.85 (0.75–0.96)**		0.90 (0.79–1.02)
Secondary and higher		0.87 (0.74–1.02)		0.94 (0.80–1.11)
Religion				
Catholics		1.00		1.00
Protestants		1.01 (0.89–1.14)		1.00 (0.88–1.13)
Muslims and other		**1.14 (1.03–1.27)**		**1.12 (1.01–1.25)**
Experienced death of child				
No		1.00		1.00
Yes		0.90 (0.55–1.47)		0.88 (0.54–1.44)
Media exposure				
No		1.00		1.00
Yes		**0.82 (0.75–0.90)**		**0.82 (0.75–0.90)**
Distance to HF				
No problem		1.00		1.00
Problem		**1.09 (1.01–1.19)**		1.05 (0.96–1.15)
*Community-level factors*				
Community wealth				
Low			1.00	1.00
Middle			0.94 (0.84–1.05)	0.90 (0.81–1.01)
High			0.87 (0.76–1.01)	**0.81 (0.67–0.96)**
Community employment				
Low			1.00	1.00
Middle			**0.87 (0.78–0.97)**	0.95 (0.85–1.06)
High			**0.76 (0.67–0.85)**	**0.88 (0.78–0.99)**
Community women’s education				
Low			1.00	1.00
Middle			**0.86 (0.77–0.98)**	**0.86 (0.76–0.96)**
High			**0.84 (0.74–0.95)**	**0.81 (0.70–0.93)**
Community distance to HF				
Low			1.00	1.00
Middle			**1.18 (1.04–1.33)**	**1.17 (1.03–1.32)**
High			1.11 (0.97–1.27)	1.08 (0.93–1.24)
Measures of variation				
Area variance (95% CI)	0.14 (0.10–0.19)	0.10 (0.06–0.15)	0.11 (0.07–0.16)	0.09 (0.05–0.14)
ICC (%)	4.0	2.9	3.2	2.7
PCV (%)	Ref.	28.6	21.4	35.7
MOR	1.43	1.35	1.37	1.33
Model Fit statistic				
AIC	16,154.26	15,877.07	16,116.63	15,860.86

Null model contains no explanatory variables; Model I includes individual-level factors only; Model II includes community-level factors only; Model III includes both individual-level and community-level factors

*aOR* adjusted odds ratio*, CI* confidence internal, *ICC* intraclass correlation coefficient, *MOR* median odds ratio, *PVC* proportional change in variance, *AIC* Akaike information criterion

### Modeling approaches (random effects)

Measures of variation for the total unmet need outcomes are displayed in Table 2. In the null models, the use of multilevel modeling was justified by the significant variation in total unmet need (σ^2^ = 0.14, 95% CI 0.10–0.20). The ICC for the total unmet need was 4.0% suggesting that variation in unmet need status may be attributable to other unobserved community characteristics. The final model revealed significant variances and the MOR of 1.33 showed the effects of community heterogeneity (i.e., suggesting that if a married woman moved to a community with a higher probability of total unmet need, the median increase in the odds of having total unmet need for FP would be 1.33-fold). Additionally, 35.7% of the variance in the odds of having total unmet need across communities explained by both individual- and community-level factors, as indicated by the PCV.

### Sensitivity analyses

After excluding those categorized as having “no unmet need”, similar results to those when these were included in the analyses were observed (Table S2). Additionally, there were 188 (1.2%) women that reported using traditional contraceptive methods. After excluding those using traditional contraceptive methods from analyses, the results were fairly consistent as those when these women were included in the total sample (Table S3). Similarly, when the analysis was restricted to married women only to control for marriage-related factors, no substantial differences from the main results except that educational level was no longer associated with unmet need (Table S4).

## Discussion

This study examined the individual- and community- level factors associated with unmet need for FP in Malawi. Apart from significant individual-level factors associated with unmet need for FP, the study also revealed significant community effects. Specifically, women from communities with a high percentage of women from rich households and from communities with a high percentage of educated women exhibited the same reduction (19.0%) in the likelihood of having total unmet need compared with those from communities with low percentages of women from rich households and educated women, respectively.

The overall prevalence for unmet need was 21.0% (a higher prevalence of unmet need for spacing (12.6%) was observed compared with 8.4% for unmet need for limiting). Among married women, the total unmet need was 18.7% representing a decline from the 26.0% unmet need prevalence among married women reported in Malawi in 2010. This reduction in unmet need in Malawi over the years could be moderately attributed to the youth-friendly FP services (under the youth friendly health services program) which has aimed at improving the usage of modern contraceptive methods among the youth. However, disparities in access to FP methods has been reported among different sociodemographic strata and understanding factors associated with unmet need in Malawi is vital for public health practitioners to design targeted, and strengthen the already existing, interventions.

### Factors associated with total unmet need

In the current analysis, married women were less likely to have total unmet need compared with unmarried women. As observed, the demand for FP was higher among sexually unmarried women compared to married women (Fig. 1 and Fig. 2). The cultural opposition to being pregnant while unmarried may raise the need for FP services among sexually active unmarried women [31]. Consistent with a study from Ghana [18], employed women were less likely to have total unmet need. Employed women may have better access to quality health services as they may be able to afford private health insurance compared with their unemployed counterparts [32]. Additionally, employed women are more likely to be independent and have better autonomy on their health decisions and therefore, may exhibit better behaviors in health services utilization (including contraceptive use) [32, 33].

Regional variations in terms of the total unmet need were observed with women from the central and southern region being less likely to have unmet need for FP. It has been reported that in Malawi, women from the northern region have the lowest rates of use of modern contraceptives [20]. The northern region is dominantly a patrilineal society hence most women depend on their partners when it comes to healthcare decisions (including FP). A Ugandan study reported that men were less likely to have knowledge of contraceptives with most of them expressing fear of the side effects of modern contraceptive methods to their partners [34]. Additionally, women in the Northern region were reported to have more co-wives compared with those from the central and southern regions [20]. A 2013 study in Northern Malawi revealed polygamy to be a driver of fertility preference disagreements which may in turn, influence unmet need for FP in this region [35]. Lastly, a 2012 longitudinal study conducted in Northern Malawi reported a high discontinuation of contraceptive use among women [36] which may highlight the effects of underlying institutional-based factors such as stock-outs. In the current analysis, women who reported to have had exposure to media at least once a week were less likely to report having the total unmet need. A study in Botswana reveled that women who reported to have been listening to radio at least once a week were less likely to have unmet need [37]. Strengthening media programs in disseminating FP messages on the importance of FP methods, and where they can be accessed is thus essential in Malawi.

Our findings revealed that women who belonged to Islam and other religions were more likely to have total unmet need for FP compared with those belonging to the Catholic religion. This is consistent with a Nepalese study in which Muslim women were positively associated with having unmet need [38]. Religious beliefs have been shown to influence health behaviors. For instance, religiosity influenced fertility preferences in the United States [39]. In Iran, fertility preferences were higher among individuals with stronger religious beliefs [40]. The variations within different religious groups observed in this study underscore the need for FP programs to engage different religious institutions and influential religious leaders to effectively scale up FP services.

The effects of community characteristics on health outcomes and behaviors have been well-documented [32, 41]. Findings from the current analysis revealed that women from communities with a high percentage of women from rich households and educated women were less likely to have total unmet need. Educated women are more likely to comprehend health messages and demand services [42]. Additionally, educated women are more likely to be empowered which may subsequently increase their contraceptive use [43, 44]. Similarly, women from rich households have better chances of accessing information and affording private health facilities to access FP services. As such, women from communities with a high percentage of rich and educated women may learn from others on the importance of using FP services and where these may be accessed. Compared with women from communities with a low percentage of women complaining of the distance to a health facility, those from communities with a middle percentage of women complaining of the distance to health facility were more likely to have unmet need for FP. In Ethiopia, proximity to a health facility was independently associated with contraceptive utilization [45]. The associations with perceived distance were more pronounced at the community level than at the individual level because in the local setting, individual effects may be attenuated by services that have been rolled out (i.e., mobile clinic services and community health workers) that mainly target individuals [9].

### Policy/program implications

First, regional differences were observed. FP programs and interventions need to be strengthened in the northern region of Malawi. Second, as observed, employed women were less likely to have total unmet need suggesting that empowering women may go a long way in addressing FP challenges. Third, there were unobserved or unmeasured community factors that influenced unmet need for FP. This highlights that there are factors operating at the community level, not included in the current analysis, which may be associated with unmet need in Malawi. These may include but are not limited to cultural differences between communities (that may ultimately influence misconceptions and myths about FP), and community outreach, engagement, and mobilization efforts. Therefore, FP programs need to conduct thorough community profiling, and strengthen their community engagement approaches involving relevant stakeholders such as community leaders and religious institutions.

### Strengths and limitations

The study included a nationally representative sample of women in Malawi therefore, results from the current analysis may be generalized to Malawian women. The hierarchical nature of the DHS dataset allowed for exploration of community effects which may have an influence on FP programming in Malawi. A wide range of factors were assessed in this study to strengthen the associations observed. The cross-sectional nature of the study means causality cannot be inferred. The use of administratively defined boundaries has the potential of introducing misclassification for unfitted administrative communities.

## Conclusion

A higher rate of unmet need for spacing (12.6.8%) was observed compared to the rate for unmet need for limiting (8.4%). In Malawi, factors influencing unmet need for FP operate at both individual and community level. FP programs in Malawi should be strengthened in disadvantaged communities, and the northern region. Qualitative research is needed in Malawi to understand some of the observations made in the current analysis and to divulge more information on the influences of cultural, and religious beliefs that may explain some of the unaccounted community effects.

## Supplementary information

**Additional file 1.** The supplement has tables s1 (multicollinearity tests), and s3- s4 (sensitivity analyses)

## Data Availability

The study used, with permission, data from the International Classification of Functioning, Disability, and Health (ICF). The data is publicly available upon request from the ICF on (https://dhsprogram.com/data/available-datasets.cfm).

## Abbreviations

aOR: Adjusted odds ratio
CI: confidence interval
MDHS: Malawi demographic health survey
FP: Family planning
ICC: Intraclass correlation coefficient
PVC: Proportional change in variance
AIC: Akaike information criterion
MOR: Median odds ratio
