# Supplementary material for: Multilevel analysis of factors associated with unmet need for family planning among Malawian women
Source: BMC Public Health. 2020 May 15;20:705. doi: 10.1186/s12889-020-08885-1 (PMC7229612; doi:10.1186/s12889-020-08885-1)
Supplement: Supplementary file 1 — Additional file 1. The supplement has tables s1 (multicollinearity tests), and s3- s4 (sensitivity analyses) [file 12889_2020_8885_MOESM1_ESM.docx]

| Table S1 Multicollinearity | |
| --- | --- |
| Variable | VIF |
| Total unmet need | 1.02 |
| Women’s age | 1.61 |
| Number of children ever had | 2.50 |
| Wealth | 1.66 |
| Employed | 1.20 |
| Residence | 1.74 |
| Region | 1.18 |
| Educational level | 1.49 |
| Religion | 1.06 |
| Ever experienced child’s death | 1.96 |
| Media exposure | 1.18 |
| Distance to health facility | 1.40 |
| Community wealth | 2.46 |
| Community employment | 1.28 |
| Community women’s education | 1.77 |
| Community distance to health facility | 1.77 |
| VIF; variance inflation factor | |

**Multilevel analysis of factors associated with unmet need for family planning among Malawian women**

Owen Nkoka, Watanja M Mphande Peter AM Ntenda, Edith B Milanzi, Victor Kanje and Gina Guo

**Online Supplement**

| Table S2 Multilevel logistic analysis of factors associated with unmet needs among sexually active women (Excluding those with “No unmet need) | | |
| --- | --- | --- |
| Variable | | Model III only  aOR (95% CI) |
| *Individual-level factors* | |  |
| Woman’s age (years) | |  |
|  | 15 – 24 | 1.00 |
|  | 25 – 34 | 1.02 (0.90 – 1.16) |
|  | ≥ 35 | 1.08 (0.94 – 1.23) |
| Marital status | |  |
|  | Unmarried | 1.00 |
|  | Married | 0.87 (0.71 – 1.06) |
| Number of children ever had | |  |
|  | 0 | 1.00 |
|  | 1 | **0.32 (0.17 – 0.61)** |
|  | 2+ | **0.27 (0.14 – 0.52)** |
| Wealth | |  |
|  | Poor | 1.00 |
|  | Middle | 0.92 (0.82 – 1.04) |
|  | Rich | 0.95 (0.84 – 1.07) |
| Employed | |  |
|  | No | 1.00 |
|  | Yes | **0.77 (0.70 – 0.85)** |
| Residence | |  |
|  | Urban | 1.00 |
|  | Rural | 0.87 (0.72 – 1.04) |
| Region | |  |
|  | Northern | 1.00 |
|  | Central | **0.65 (0.55 – 0.76)** |
|  | Southern | **0.78 (0.67 – 0.90)** |
| Woman’s educational level | |  |
|  | No formal education | 1.00 |
|  | Primary | 0.90 (0.79 – 1.03) |
|  | Secondary and higher | 0.92 (0.78 – 1.29) |
| Religion | |  |
|  | Catholics | 1.00 |
|  | Protestants | 0.99 (0.87 – 1.13) |
|  | Muslims and other | **1.15 (1.03 – 1.29)** |
| Experienced death of child | |  |
|  | No | 1.00 |
|  | Yes | **2.52 (1.38 – 4.60)** |
| Media exposure | |  |
|  | No | 1.00 |
|  | Yes | **0.78 (0.71 – 0.86)** |
| Distance to HF | |  |
|  | No problem | 1.00 |
|  | Problem | 1.05 (0.96 – 1.17) |
| *Community-level factors* | |  |
| Community wealth | |  |
|  | Low | 1.00 |
|  | Middle | 0.91 (0.80 – 1.04) |
|  | High | **0.85 (0.64 – 0.97)** |
| Community employment | |  |
|  | Low | 1.00 |
|  | Middle | 0.94 (0.83 – 1.07) |
|  | High | **0.85 (0.74 – 0.97)** |
| Community women’s education | |  |
|  | Low | 1.00 |
|  | Middle | **0.79 (0.69 – 0.90)** |
|  | High | **0.75 (0.64 – 0.88)** |
| Community distance to HF | |  |
|  | Low | 1.00 |
|  | Middle | **1.18 (1.03 – 1.35)** |
|  | High | 1.10 (0.93 – 1.28) |
| Measures of variation | |  |
|  | Area variance (95% CI) | 0.15 (0.11 – 0.22) |
|  | ICC (%) | 4.5 |
|  | PCV (%) | 31.8 |
|  | MOR | 1.46 |
| Model Fit statistic | |  |
|  | AIC | 14098.10 |
| Model III includes both individual-level and community-level factors  aOR adjusted odds ratio, CI confidence internal, ICC intraclass correlation coefficient, MOR median odds ratio, PVC proportional change in variance, AIC Akaike information criterion | | |

| Table S3 Multilevel logistic analysis of factors associated with unmet needs among sexually active women (Modern contraceptives only) | | |
| --- | --- | --- |
| Variable | | Model III only  aOR (95% CI) |
| *Individual-level factors* | |  |
| Woman’s age (years) | |  |
|  | 15 – 24 | 1.00 |
|  | 25 – 34 | 0.99 (0.88 – 1.11) |
|  | ≥ 35 | **1.21 (1.06 – 1.38)** |
| Marital status | |  |
|  | Unmarried | 1.00 |
|  | Married | **0.40 (0.34 – 0.47)** |
| Number of children ever had | |  |
|  | 0 | 1.00 |
|  | 1 | **0.60 (0.36 – 0.99)** |
|  | 2+ | 0.60 (0.36 – 1.02) |
| Wealth | |  |
|  | Poor | 1.00 |
|  | Middle | 0.95 (0.85 – 1.06) |
|  | Rich | 0.99 (0.88 – 1.11) |
| Employed | |  |
|  | No | 1.00 |
|  | Yes | **0.78 (0.71 – 0.85)** |
| Residence | |  |
|  | Urban | 1.00 |
|  | Rural | 0.91 (0.77 – 1.07) |
| Region | |  |
|  | Northern | 1.00 |
|  | Central | **0.68 (0.59 – 0.78)** |
|  | Southern | **0.79 (0.69 – 0.90)** |
| Woman’s educational level | |  |
|  | No formal education | 1.00 |
|  | Primary | 0.90 (0.79 – 1.02) |
|  | Secondary and higher | 0.95 (0.80 – 1.11) |
| Religion | |  |
|  | Catholics | 1.00 |
|  | Protestants | 1.01 (0.89 – 1.14) |
|  | Muslims and other | **1.13 (1.01 – 1.25)** |
| Experienced death of child | |  |
|  | No | 1.00 |
|  | Yes | 0.87 (0.53 – 1.43) |
| Media exposure | |  |
|  | No | 1.00 |
|  | Yes | **0.83 (0.75 – 0.90)** |
| Distance to HF | |  |
|  | No problem | 1.00 |
|  | Problem | 1.05 (0.96 – 1.15) |
| *Community-level factors* | |  |
| Community wealth | |  |
|  | Low | 1.00 |
|  | Middle | 0.90 (0.80 – 1.01) |
|  | High | **0.80 (0.67 – 0.96)** |
| Community employment | |  |
|  | Low | 1.00 |
|  | Middle | 0.94 (0.84 – 1.06) |
|  | High | 0.89 (0.78 – 1.01) |
| Community women’s education | |  |
|  | Low | 1.00 |
|  | Middle | **0.86 (0.76 – 0.96)** |
|  | High | **0.81 (0.70 – 0.93)** |
| Community distance to HF | |  |
|  | Low | 1.00 |
|  | Middle | **1.17 (1.03 – 1.33)** |
|  | High | 1.08 (0.93 – 1.25) |
| Measures of variation | |  |
|  | Area variance (95% CI) | 0.10 (0.06 – 0.15) |
|  | ICC (%) | 2.9 |
|  | PCV (%) | 28.6 |
|  | MOR | 1.35 |
| Model Fit statistic | |  |
|  | AIC | 15761.72 |
| Model III includes both individual-level and community-level factors  aOR adjusted odds ratio, CI confidence internal, ICC intraclass correlation coefficient, MOR median odds ratio, PVC proportional change in variance, AIC Akaike information criterion | | |

| Table S4 Multilevel logistic analysis of factors associated with unmet needs among sexually active women (Married women only) | | |
| --- | --- | --- |
| Variable | | Model III only  aOR (95% CI) |
| *Individual-level factors* | |  |
| Woman’s age (years) | |  |
|  | 15 – 24 | 1.00 |
|  | 25 – 34 | 0.97 (0.86 – 1.10) |
|  | ≥ 35 | **1.17 (1.02 – 1.34)** |
| Age at first marriage (years) | |  |
|  | ≤ 24 | 1.00 |
|  | 25 – 24 | 1.22 (0.99 – 1.51) |
|  | ≥ 35 | 1.30 (0.68 – 2.47) |
| Number of children ever had | |  |
|  | 0 | 1.00 |
|  | 1 | 0.77 (0.43 – 1.36) |
|  | 2+ | 0.78 (0.43 – 1.40) |
| Wealth | |  |
|  | Poor | 1.00 |
|  | Middle | 0.93 (0.83 – 1.04) |
|  | Rich | 0.95 (0.84 – 1.07) |
| Employed | |  |
|  | No | 1.00 |
|  | Yes | **0.81 (0.74 – 0.90)** |
| Residence | |  |
|  | Urban | 1.00 |
|  | Rural | 0.96 (0.81 – 1.15) |
| Region | |  |
|  | Northern | 1.00 |
|  | Central | **0.68 (0.59 – 0.79)** |
|  | Southern | **0.80 (0.70 – 0.92)** |
| Woman’s educational level | |  |
|  | No formal education | 1.00 |
|  | Primary | 0.91 (0.79 – 1.04) |
|  | Secondary and higher | 0.92 (0.77 – 1.09) |
| Religion | |  |
|  | Catholics | 1.00 |
|  | Protestants | 1.00 (0.88 – 1.14) |
|  | Muslims and other | **1.13 (1.01 – 1.26)** |
| Partner’s educational level | |  |
|  | No formal education | 1.00 |
|  | Primary | 0.87 (0.76 – 1.01) |
|  | Secondary and higher | 0.97 (0.82 – 1.14) |
| Experienced death of child | |  |
|  | No | 1.00 |
|  | Yes | 0.81 (0.47 – 1.41) |
| Media exposure | |  |
|  | No | 1.00 |
|  | Yes | **0.83 (0.75 – 0.90)** |
| Distance to HF | |  |
|  | No problem | 1.00 |
|  | Problem | 1.06 (0.96 – 1.17) |
| *Community-level factors* | |  |
| Community wealth | |  |
|  | Low | 1.00 |
|  | Middle | 0.93 (0.82 – 1.04) |
|  | High | **0.83 (0.69 – 1.00)** |
| Community employment | |  |
|  | Low | 1.00 |
|  | Middle | 0.93 (0.83 – 1.04) |
|  | High | 0.90 (0.79 – 1.02) |
| Community women’s education | |  |
|  | Low | 1.00 |
|  | Middle | **0.88 (0.77 – 0.99)** |
|  | High | **0.83 (0.71 – 0.97)** |
| Community men’s education | |  |
|  | Low | 1.00 |
|  | Middle | 0.94 (0.83 – 1.06) |
|  | High | 0.99 (0.86 – 1.14) |
| Community distance to HF | |  |
|  | Low | 1.00 |
|  | Middle | 1.13 (0.99 – 1.28) |
|  | High | 1.07 (0.92 – 1.24) |
| Measures of variation | |  |
|  | Area variance (95% CI) | 0.10 (0.06 – 0.15) |
|  | ICC (%) | 2.8 |
|  | PCV (%) | 33.3 |
|  | MOR | 1.34 |
| Model Fit statistic | |  |
|  | AIC | 14818.44 |
|  | | |
